# AOA-Assisted TDOA Localization Based on Improved Whale Optimization Algorithm for Asynchronous Wireless System Networks

**DOI:** 10.3390/s26175442

**Published:** 2026-08-28

**Authors:** Liang Qi, Zhiyong Liu, Mude Cai, Liangbo Xie

**Affiliations:** 1 School of Electronic Information Engineering, Nanjing University of Aeronautics and Astronautics, Nanjing 210016, China; ql15380391990@126.com; 2 No. 723 Institute of China State Shipbuilding Co., Ltd., Yangzhou 225101, China; qcdzyzhiyong@163.com; 3 School of Communications and Information Engineering, Chongqing University of Posts and Telecommunications, Chongqing 400065, China; s240131108@stu.cqupt.edu.cn

**Keywords:** WSN, localization, TDOA, AOA, relative clock offset and skew estimation

## Abstract

This paper proposes a AOA-assisted TDOA localization method based on an improved whale optimization algorithm (IWOA) combining TDOA and AOA for asynchronous wireless sensor networks (WSNs). A TDOA compensation scheme is first introduced to address network asynchrony. This scheme uses the communication timestamps between anchor nodes to estimate relative clock deviations and clock offsets. Then, a fusion algorithm using the compensated TDOA and AOA measurements enables high-precision localization with only two angle-measuring anchor nodes and a set of TDOA anchor nodes, reducing the number of required anchor nodes and improving deployment flexibility. Finally, the integration of IWOA enhances the optimization process, improving robustness. Simulations demonstrate that the proposed IWOA achieves superior robustness and accuracy under severe noise and adverse geometric conditions, significantly outperforming the Chan algorithm and other metaheuristic benchmarks. Meanwhile, the CWLS algorithm exhibits competitive performance in well-calibrated scenarios, revealing their complementary characteristics. The proposed method provides an effective solution for asynchronous WSNs, especially in challenging measurement environments.

## 1. Introduction

Wireless Sensor Networks (WSNs), as a core component of the Internet of Things, have demonstrated broad application prospects in recent years across areas such as environmental monitoring, smart homes, industrial automation, and emergency rescue [1,2,3,4,5,6]. In these applications, obtaining precise location information from network nodes is critical, such as quickly locating trapped individuals during disaster relief or tracking goods in real time in smart warehouse systems [7,8]. Therefore, achieving reliable and high-precision positioning of target nodes has become a key issue in advancing the practical application of WSN-related technologies [9,10].

Generally, positioning methods in WSNs primarily include Time of Arrival (TOA) [11,12,13], Time Difference of Arrival (TDOA) [14,15,16], Angle of Arrival (AOA) [17,18] and Received Signal Strength Indicator (RSSI) [19]. Among these, TOA achieves high-precision positioning by measuring signal propagation distances, but relies on strict clock synchronization between nodes [11,12]. The angle sensor used by the AOA sensors are expensive. The RSSI is easily affected by the environment and has poor positioning accuracy. In contrast, TDOA does not require strict synchronization [15,16]. Therefore, positioning algorithm based on TDOA information has received wider attention.

However, TDOA performance is still constrained by synchronization accuracy between anchor nodes and the number of anchor nodes available [16]. Various algorithms have been developed to address the clock synchronization problem in WSNs. In early studies, clock synchronization was typically regarded as an independent preprocessing step. For example, a belief propagation scheme (BP) based on factor graph (FG) aims to reduce the synchronization overhead through iterative optimization [20,21]. However, synchronization errors can be propagated directly into positioning errors, thereby constraining the overall accuracy of the system [22]. To tackle this problem, recent research trends have gradually shifted toward jointly solving synchronization and localization in a coupled manner. One joint estimation framework leverages periodic broadcast signals from anchor nodes: it first estimates relative clock offsets through TDOA measurements and then performs position calculation based on the compensated time differences. This stepwise optimization strategy significantly improves the robustness of the system [22,23]. On the other hand, for dynamic scenarios and high-frequency mobile devices, such as device localization in 5G technology [24], researchers have further developed joint estimation algorithms based on the extended Kalman filter (EKF) [25]. This approach not only accounts for the nonlinear variations in positional states but also simultaneously estimates clock drift and jitter, thereby maintaining high-precision positioning performance even in environments with pronounced Doppler effects.

In addition, researchers have proposed hybrid positioning algorithms to improve positioning accuracy. Integrating multiple types of measurement information to enhance positioning performance has become an important research direction [26,27]. For example, the hybrid TDOA/AOA method, by jointly utilizing time difference and angle of arrival information, can improve system robustness under varying working conditions [28,29]. This approach not only alleviates the dependency on the number of anchor nodes and reduces the required TDOA measurements, but also effectively suppresses the impact of single-measurement errors on positioning results through complementary information [26,28]. Thus, for asynchronous or weakly synchronized WSN scenarios, developing high-performance TDOA/AOA fusion positioning algorithms has significant theoretical and practical value [29,30].

Modeling and solving the joint localization problem present significant technical challenges. First, the AOA algorithm calculates the position of the target node using the line connecting the target node to the anchor nodes, while TDOA establishes a hyperbolic measurement model [31,32]. In the joint model, the nonlinear characteristics of the two methods interact, producing a highly non-convex objective function with many local optima [31]. Second, factors such as environmental noise, hardware latency, and synchronization errors severely degrade the accuracy of both AOA and TDOA measurements, further amplifying model uncertainty [33,34,35]. Finally, joint localization involves the optimization of high-dimensional parameters, which requires ensuring the physical feasibility of the estimated results while satisfying constraints related to sensor spatial positions and observation values [29,36].

This paper focuses on the TDOA/AOA positioning problem in asynchronous WSN environments, addressing shortcomings in existing algorithms such as clock synchronization compensation and joint optimization of measurement errors. The main innovations are summarized in the following three aspects:

- To address the inherent asynchrony in wireless sensor networks, this paper introduces a TDOA calibration method based on relative clock parameter estimation. By employing periodic inter-anchor communication, the relative clock offsets and skews among anchor nodes are accurately estimated, thereby achieving effective relative synchronization. This approach substantially mitigates the adverse effects of network asynchrony on conventional TDOA-based localization, providing a reliable foundation for subsequent positioning stages.
- Based on the calibrated TDOA measurements, a AOA-assisted TDOA localization algorithm integrating TDOA and AOA measurements is developed. The proposed model only requires two anchor nodes to have angle measurement capabilities, while the other anchor nodes measure TDOA information, enabling positioning. This model significantly reduces hardware requirements and improves the system’s applicability in practical wireless sensor network deployments.
- To address the non-convex optimization problem posed by the TDOA/AOA model, an improved whale optimization algorithm (IWOA) is applied for position estimation. The IWOA initializes its population using coarse positioning results derived from AOA measurements and iteratively refines the solution by minimizing TDOA residuals. Comprehensive simulations confirm that the proposed IWOA-based method outperforms traditional approaches, such as Chan’s algorithm, and other metaheuristic algorithms in both localization accuracy and convergence stability under diverse operating conditions.

The structure of this paper is organized as follows: The introduction is provided in Section 1. The system model is given in Section 2. The proposed estimation approach for the relative clock offsets and skews and the compensation of the original TDOA is introduced in Section 3. The TDOA/AOA fusion positioning algorithm is presented in Section 4. The simulation results are given in Section 5. Section 6 concludes this work.

## 2. System Model

**Figure 1:**
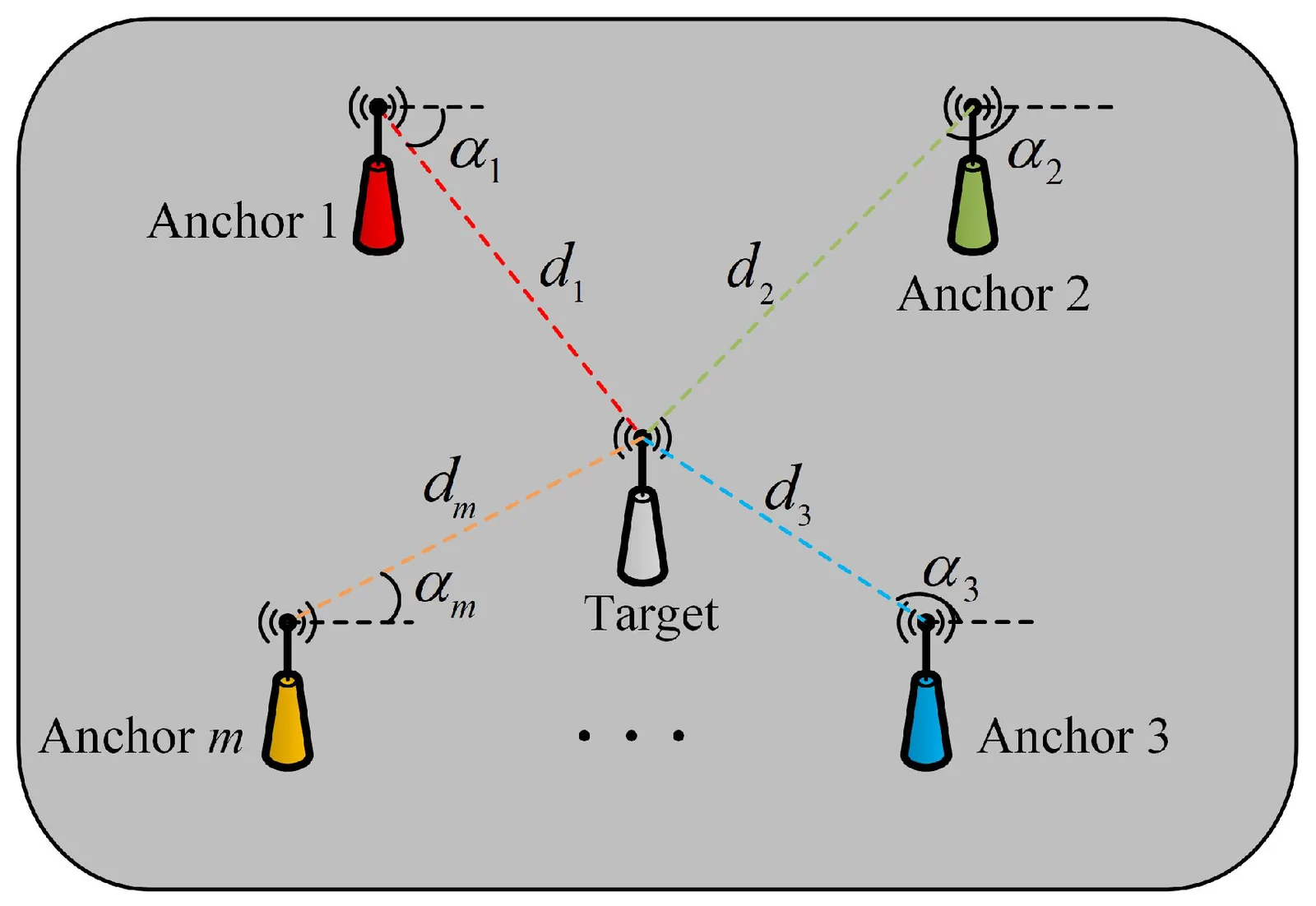
WSN Localization system model (the anchor nodes estimates that distances and angles to the target are $d_{1}$–$d_{m}$ and $\alpha_{1}$–$\alpha_{m}$, respectively).

In a two-dimensional WSN localization system, the fundamental structure of the positioning model is illustrated in Figure 1. Sensor nodes are typically categorized into anchor nodes and target nodes. In this study, anchor nodes function as asynchronous sensors and serve as reference points, with their positions pre-deployed and fixed prior to system operation. Target nodes are the objects to be localized. The network is assumed to contain *m* anchor nodes, where m≥4. For simplicity, this work considers only a single target node. When multiple target nodes are present, a typical strategy is employed to avoid communication conflicts: introducing a small random time offset to each target’s scheduled transmission time relative to a common reference.

**Figure 2:**
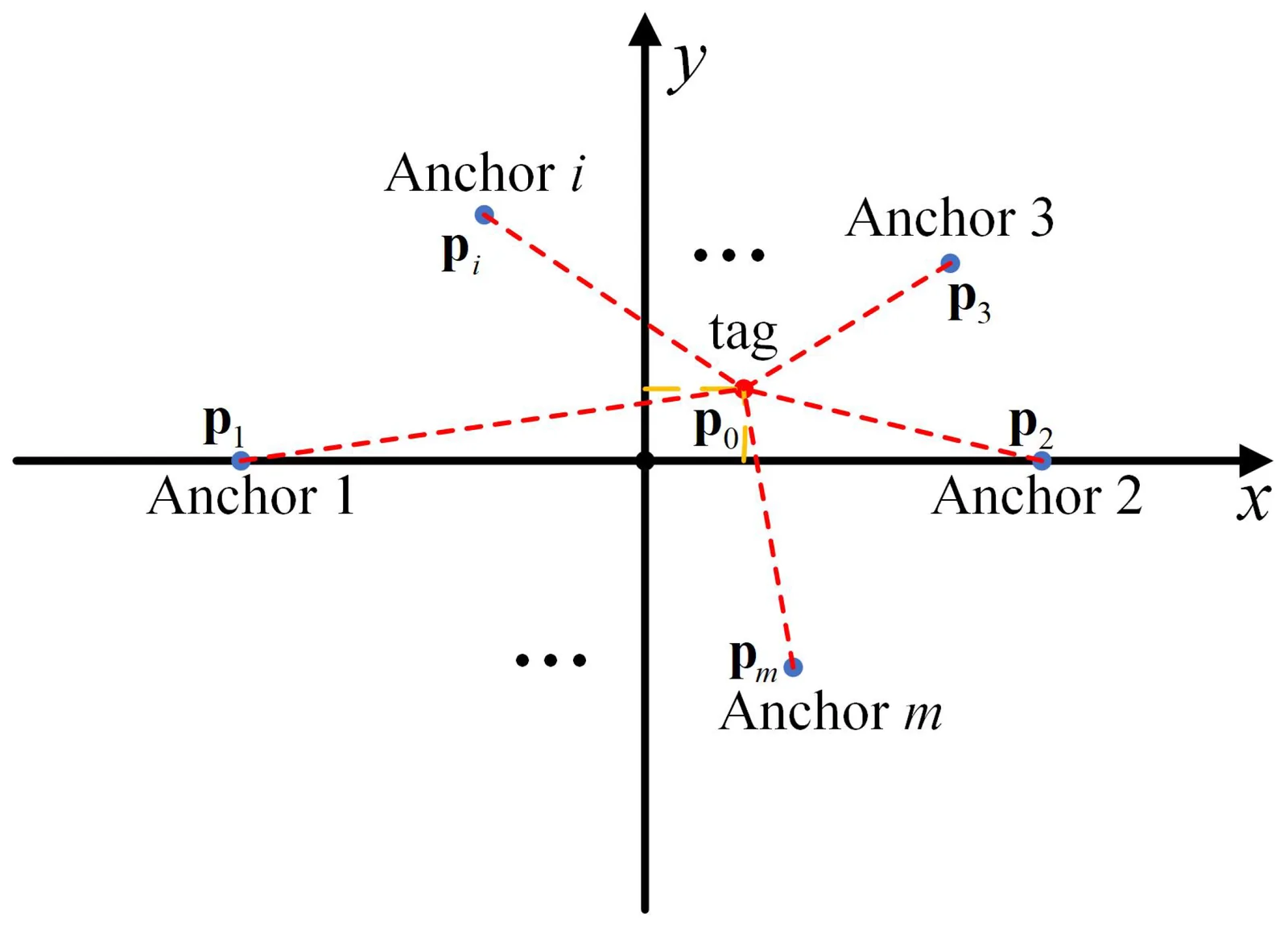
Relative coordinate system of anchor nodes and tags.

In practical engineering applications, the accurate relative positions of anchor nodes are of critical importance, and the geographic positions of anchor nodes can be obtained through coordinate transformation. For the relative positioning, it is necessary to rely on a reference coordinate system to complete the positioning and calibration of all anchor nodes. Here we consider the 2D plane as shown in Figure 2. Define the position coordinate vector of Anchor *i* as $p_{i}=\left[x_{i},y_{i}\right]$, where, i=1,2,…,m and the position coordinate vector of the target as $p_{0}=\left[x_{0},y_{0}\right]$.

To construct a relative coordinate system, we need to preset the reference origin and the baseline directions of the X-axis and Y-axis. Without loss of generality, the midpoint of the line connecting Anchor 1 and Anchor 2 is selected as the coordinate origin; the line connecting Anchor 1 and Anchor 2 serves as the baseline for the X-axis, with Anchor 1 located on the positive semi-axis of the X-axis. The Y-axis is set as the coordinate axis passing through the origin and perpendicular to the baseline direction of the X-axis. To eliminate the ambiguity of the positive direction of the Y-axis, it is assumed that the y-coordinate of Anchor 3 is positive. Based on the above settings, the construction of the relative coordinate system is completed.

**Figure 3:**
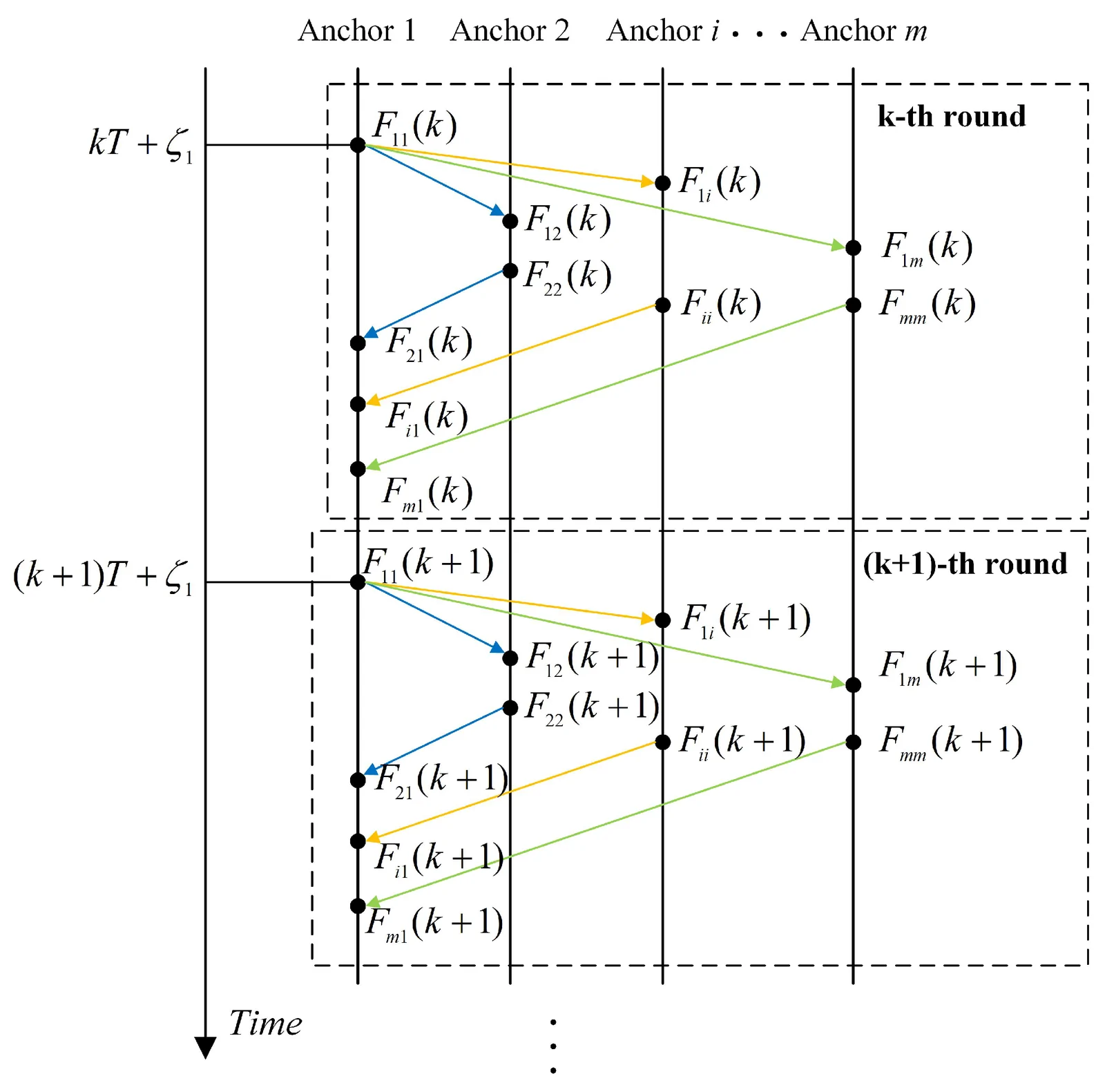
Two consecutive rounds of communication between anchor nodes.

**Figure 4:**
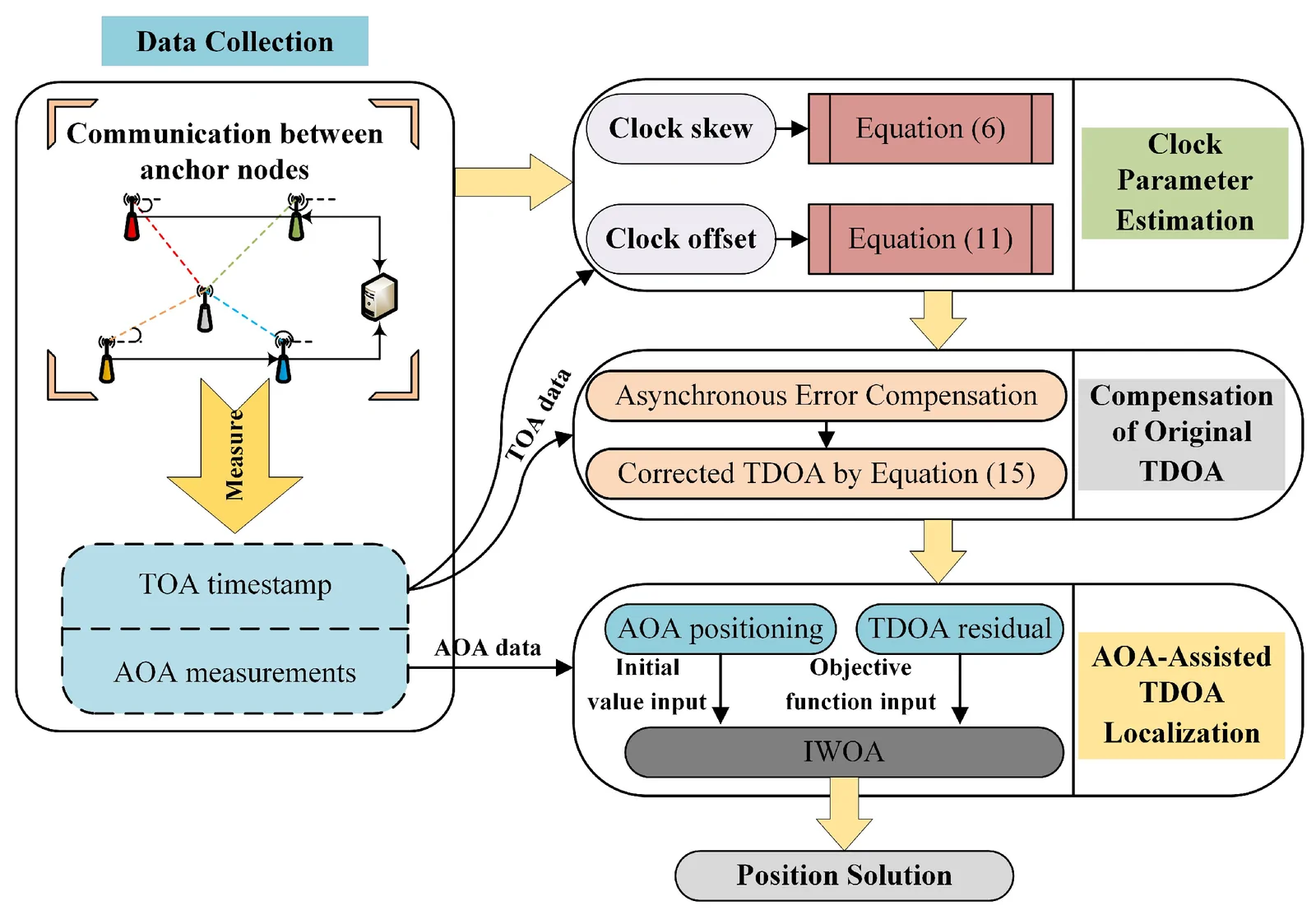
The flowchart of AOA-assisted TDOA localization.

The workflow of an anchor node is depicted in Figure 3. During the localization process, Anchor 1 periodically broadcasts broadcasts signals containing its own location every *T* seconds. Other anchor nodes receive these signals, record the TOA and subsequently return response signals containing their own locations. Anchor 1 records the TOA of these response signals based on its local clock. The target continuously broadcasts signals to all anchor nodes, which receive and record both the TOA and AOA of these signals. All TOA and AOA information is transmitted to the target node for further processing. Using the TOA measurements recorded between anchor nodes, the relative clock offsets and skews among them can be estimated. Subsequently, these estimated clock parameters are used to compensate for the original TOA measurements from the target, enabling the calculation of the corrected TDOA. Finally, the target’s location is determined by fusing the AOA measurements with the corrected TDOA. The flowchart for AOA-assisted TDOA localization is shown in Figure 4. Given the higher hardware requirements for AOA measurement and the need to reduce implementation complexity, this paper specifies that only Anchor 1 and Anchor 2 are equipped with angle-measurement capabilities.

In general, TDOA-based localization algorithms require estimation of relative clock offsets or synchronization among anchor nodes. Utilizing the inter-anchor TOA timestamps and the target-to-anchor TOA information, the steps of the proposed AOA-assisted TDOA localization algorithm are summarized as follows: (1) estimate the relative clock skews among anchor nodes using TOA measurements (Section 3.1); (2) estimate the relative clock offsets among anchor nodes using the results from the previous step and TOA measurements (Section 3.2); (3) correct the raw TDOA using the estimated clock parameters (Section 3.3); (4) fuse the corrected TDOA with AOA measurements and solve the location via an optimization algorithm (Section 4).

The proposed algorithm does not require any prior information from external precision clock devices. This paper adopts an affine clock model [23,37,38]. It is assumed that the local clock of any node (anchor or target) is imperfect, causing its local time to deviate from the ideal reference time. Therefore, the local time of node *i* can be modeled as:

(1) $$T_{i}\left(t\right)=\left(1+\varepsilon_{i}\right)t+\zeta_{i},$$

where $T_{i}\left(t\right)$ and *t* represent the local time and ideal time of anchor i(i=1,2,…,m), respectively; $\varepsilon_{i}$ is the clock skew, and $\zeta_{i}$ is the clock offset. Assuming anchor 1’s clock is the reference clock, so that $\varepsilon_{1}=\zeta_{1}=0$.

Additionally, this paper adopts three assumptions regarding the localization in WSNs [23]:

- The sensor node has a coverage range of 5000 m, which is the maximum range for the medium-range radiotransmitter in the ISM (Industry, Science, and Medicine) band, such as IEEE 802.15.4g and NB-IoT [39,40].
- There are low-cost quartz oscillators in all sensor nodes, and the typical clock skew is less than 10 ppm (parts per million), i.e., $|\varepsilon_{i}|\;\leq 10^{-5}$.
- Suppose the anchor nodes are adequately powered and have the ability to transmit data to the target node. The target node has enough computational capability.

## 3. Calibration of TDOA Based on Clock Parameter Estimation

### 3.1. Estimation of Relative Anchor Clock Skews

Based on the assumptions of the WSN, the clocks used by each anchor node are generally stable enough to meet the accuracy requirements for pulse repetition period measurements. Assuming that each anchor node locally generates periodic pulse signals with the same period, the pulse repetition periods of all anchor nodes can be approximated as a fixed value. The clock relationships among the anchor nodes can be established by utilizing these periodic pulse signals. Let this pulse repetition period be equal to *T*, the difference between the timestamps of two consecutive reception periods at the Anchor *i* can be expressed as

(2) $$\begin{array}{c} T_{i}\left(\left(k+1\right)T\right)-T_{i}\left(kT\right)=\left(1+\varepsilon_{i}\right)T, \end{array}$$

Then,

(3) $$T=\frac{T_{i}\left(\left(k+1\right)T\right)-T_{i}\left(kT\right)}{1+\varepsilon_{i}},$$

where $T_{i}\left(\left(k+1\right)T\right)$ and $T_{i}\left(kT\right)$ denote the timestamps of two consecutive receptions of pulse signals at Anchor i(i=1,2,…,m). Furthermore, the clock of Anchor 1 is taken as the reference clock. In particular,

(4) $$T=T_{1}\left(\left(k+1\right)T\right)-T_{1}\left(kT\right).$$

Taking (4) into (3):

(5) $$\frac{T_{i}\left(\left(k+1\right)T\right)-T_{i}\left(kT\right)}{1+\varepsilon_{i}}=T_{1}\left(\left(k+1\right)T\right)-T_{1}\left(kT\right),$$

then

(6) $$\varepsilon_{i}=\frac{T_{i}\left(\left(k+1\right)T\right)-T_{i}\left(kT\right)}{T_{1}\left(\left(k+1\right)T\right)-T_{1}\left(kT\right)}-1.$$

### 3.2. Estimation of Relative Anchor Clock Offsets

As shown in Figure 3, anchor 1 broadcasts signals at intervals of period *T*. The remaining anchor nodes receive the signal and return acknowledgments. Anchor 1 receives all acknowledgment signals. All anchor nodes record the round numbers and timestamps of all transmitted or received signals. This information is transmitted to the target node for estimation of clock parameters. In the *k*-th round of broadcasting, Anchor 1 broadcasts the signal at time $F_{11}\left(k\right)$. Anchor node *i* receives this signal at time $F_{1i}\left(k\right)$ relative to its local clock and replies with an acknowledgment signal at time $F_{ii}\left(k\right)$. Anchor node 1 receives the acknowledgment signal at time $F_{i1}\left(k\right)$. Communication interactions in each round should be independent; therefore, it is assumed that $T\gg \max_{i}\left(F_{i1}\left(k\right)-F_{11}\left(k\right)\right)$. According to (1), it is assumed that the timestamps for communication between the aforementioned anchor nodes can be expressed as

(7) $$\left\{\begin{array}{cc} F_{11}\left(k\right)= & \left(1+\varepsilon_{1}\right)l_{1}\left(k\right)+\zeta_{1}\\ F_{1i}\left(k\right)= & \left(1+\varepsilon_{i}\right)l_{2}\left(k\right)+\zeta_{i}\\ F_{ii}\left(k\right)= & \left(1+\varepsilon_{i}\right)l_{3}\left(k\right)+\zeta_{i}\\ F_{i1}\left(k\right)= & \left(1+\varepsilon_{1}\right)l_{4}\left(k\right)+\zeta_{1} \end{array}\right.,$$

where $l_{1}\left(k\right),l_{2}\left(k\right),l_{3}\left(k\right)$ and $l_{4}\left(k\right)$ represent the true times corresponding to the timestamps $F_{11}\left(k\right),F_{1i}\left(k\right),F_{ii}\left(k\right)$ and $F_{i1}\left(k\right)$, respectively. Since the clock of Anchor 1 is taken as reference clock, (7) can be simplified to

(8) $$\left\{\begin{array}{cc} F_{11}\left(k\right)= & l_{1}\left(k\right)\\ F_{1i}\left(k\right)= & \left(1+\varepsilon_{i}\right)l_{2}\left(k\right)+\zeta_{i}\\ F_{ii}\left(k\right)= & \left(1+\varepsilon_{i}\right)l_{3}\left(k\right)+\zeta_{i}\\ F_{i1}\left(k\right)= & l_{4}\left(k\right) \end{array}\right..$$

Assuming that the propagation delay between Anchor 1 and Anchor *i* in the *k*-th round is denoted as $\tau_{i}\left(k\right)$, the transmission equation can be expressed as

(9) $$\left\{\begin{array}{c} l_{1}\left(k\right)+\tau_{i}\left(k\right)=l_{2}\left(k\right)\\ l_{3}\left(k\right)+\tau_{i}\left(k\right)=l_{4}\left(k\right) \end{array}\right..$$

Since a sufficiently small $\varepsilon_{i}$ (where $|\varepsilon_{i}|\;\leq 10^{-5}$ ), it follows from Taylor’s formula that $\frac{1}{1+\varepsilon_{i}}=1-\varepsilon_{i}$. Taking (8) into (9):

(10) $$\left\{\begin{array}{c} F_{11}\left(k\right)+\tau_{i}\left(k\right)+\zeta_{i}\left(1-\varepsilon_{i}\right)-F_{1i}\left(k\right)\cdot \left(1-\varepsilon_{i}\right)=0\\ F_{i1}\left(k\right)-\tau_{i}\left(k\right)+\zeta_{i}\left(1-\varepsilon_{i}\right)-F_{ii}\left(k\right)\cdot \left(1-\varepsilon_{i}\right)=0 \end{array}\right..$$

The clock offset of Anchor *i* relative to Anchor 1 is solved as:

(11) $$\zeta_{i}\dot{=}\frac{\left(F_{1i}\left(k\right)+F_{ii}\left(k\right)\right)\left(1-\varepsilon_{i}\right)-F_{11}\left(k\right)-F_{i1}\left(k\right)}{2(1-\varepsilon_{i})}.$$

Therefore, in the *k*-th round, only the four corresponding timestamps are required to estimate the clock deviation of the Anchor *i*.

### 3.3. Compensation of Original TDOA

Assuming the target broadcasts a TOA signal at time $t_{0}$ of the reference clock, and Anchor 1 and *i* record local timestamps $T_{1}\left(t_{1}\right)$ and $T_{i}\left(t_{i}\right)$ at reference clock times $t_{1}$ and $t_{i}$, respectively, the distance difference between Anchor *i* and Anchor 1 in the asynchronous scenario can be expressed as:

(12) $$d_{i}-d_{1}=c\left[\left(t_{i}-t_{0}\right)-\left(t_{1}-t_{0}\right)\right]=c\left(t_{i}-t_{1}\right),$$

where *c* denotes the propagation velocity of the signal in vacuum, which is generally the speed of light; $t_{i}$ represents the actual TOA value of the signal transmitted from the target node to Anchor *i*. According to (1), (12) is further written as

(13) $$\begin{array}{cc} d_{i}-d_{1}= & c(\frac{T_{i}\left(t_{i}\right)-\zeta_{i}}{1+\varepsilon_{i}}-T_{1}\left(t_{1}\right))\\ \dot{=} & c[T_{i}\left(t_{i}\right)-T_{1}\left(t_{1}\right)-\zeta_{i}\left(1-\varepsilon_{i}\right)-T_{i}\left(t_{i}\right)\varepsilon_{i}], \end{array}$$

where $T_{i}\left(t_{i}\right)$ is the measured TOA value of Anchor *i*, then

(14) $$c\left[T_{i}\left(t_{i}\right)-T_{1}\left(t_{1}\right)\right]=d_{i}-d_{1}+c\zeta_{i}\left(1-\varepsilon_{i}\right)+cT_{i}\left(t_{i}\right)\varepsilon_{i}.$$

Extending (14) to m−1 groups and expressing it in vector form:

(15) $$r=d+F\zeta_{A}+\eta_{A}=d+n,$$

where $r=c\cdot {\left[T_{2}\left(t_{2}\right)-T_{1}\left(t_{1}\right),\ldots ,T_{m}\left(t_{m}\right)-T_{1}\left(t_{1}\right)\right]}^{T}$ is the TDOA measurement vector; $d={\left[d_{2}-d_{1},\ldots ,d_{m}-d_{1}\right]}^{T}$ is the true distance difference vector; $\zeta_{A}={\left[\zeta_{2},\ldots ,\zeta_{m}\right]}^{T}$ represents the estimated clock offset vector; $\eta_{A}=c\cdot {\left[T_{i}\left(t_{i}\right)\varepsilon_{i},\ldots ,T_{i}\left(t_{i}\right)\varepsilon_{i}\right]}^{T}$ is the asynchronous deviation error vector; $n=F\zeta_{A}+\eta_{A}$ is the noise vector; matrix F is a matrix of offset error coefficients (m−1)×(m−1):

(16) $$F=\left[\begin{array}{cccc} c(1-\varepsilon_{2}) & 0 & \ldots & 0\\ 0 & c(1-\varepsilon_{3}) & \ldots & 0\\ \vdots & \vdots & \ddots & \vdots \\ 0 & 0 & \ldots & c(1-\varepsilon_{m}) \end{array}\right],$$

Therefore, Equation (14) transforms asynchronous TDOA into a conventional TDOA expression, which can be solved using conventional methods. However, since the noise vector no longer adheres to a Gaussian distribution, conventional approaches may not yield the optimal solution. To enhance the final positioning accuracy, this paper adopts an optimization algorithm combined with AOA positioning for the final solution. The details are introduced in Section 4.

## 4. Optimization of AOA-Assisted TDOA Localization

The steps of the positioning optimization algorithm are as follows: first, preliminary AOA positioning is employed to achieve fast computation and quickly narrow down the possible target node area. This addresses issues such as slow global search, susceptibility to local optima, and divergence that may arise in TDOA positioning. Next, using the calibrated TDOA algorithm from the previous section, the measurement errors introduced by asynchrony between anchor nodes are inferred through clock parameter calculations. Finally, an improved whale optimization algorithm is used to iteratively optimize the theoretical TDOA residuals based on all coarse positioning results, thereby searching for the optimal solution.

Based on the system model in Section 2, only Anchor 1 and Anchor 2 can measure angle. From the true AOA of anchor j(j=1,2), it can be expressed that

(17) $$\tan\;\theta_{j}=\frac{y_{j}-y_{0}}{x_{j}-x_{0}}.$$

The measured AOA can be derived that

(18) $${\tilde{\theta }}_{j}=\theta_{j}+e_{aoa,j},$$

where $e_{aoa,j}$ represents the AOA measurement error, which is Gaussian noise with a mean of 0 and a variance of $\sigma_{aoa,j}$. Assume $\sigma_{aoa,1}=\sigma_{aoa,2}=\sigma_{aoa}$.

The unit direction vector ${\mathrm{dir}}_{j}$ of anchor *j* is solely determined by the azimuth angle:

(19) $${\mathrm{dir}}_{j}=\left[\cos\;\theta_{j},\sin\;\theta_{j}\right].$$

Taking anchor *j* as the starting point and the unit direction vector as the ray direction, the parametric equations of the two rays are obtained:

(20) $$\left\{\begin{array}{c} b_{1}\left(a_{1}\right)=p_{1}+a_{1}\cdot {\mathrm{dir}}_{1}\\ b_{2}\left(a_{2}\right)=p_{2}+a_{2}\cdot {\mathrm{dir}}_{2} \end{array}\right..$$

where $p_{1},p_{2}$ are the coordinates of two angle-measuring anchors; $a_{1},a_{2}\in R$ are the ray parameters, representing the distance from $b_{1}$ to $p_{1}$ and $b_{2}$ to $p_{2}$.

The midpoint of the line connecting the two closest points on the two rays is taken as the AOA positioning result. Define the distance squared:

(21) $$D\left(a_{1},a_{2}\right)={∥p_{1}+a_{1}{\mathrm{dir}}_{1}-\left(p_{2}+a_{2}{\mathrm{dir}}_{2}\right)∥}^{2}.$$

Take the partial derivative with respect to $a_{1}$:

(22) $$\frac{\partial D}{\partial a_{1}}=2{\mathrm{dir}}_{1}^{T}\left(p_{1}-p_{2}+a_{1}{\mathrm{dir}}_{1}-a_{2}{\mathrm{dir}}_{2}\right)=0,$$

then,

(23) $$\left({\mathrm{dir}}_{1}\cdot {\mathrm{dir}}_{1}\right)a_{1}-\left({\mathrm{dir}}_{1}\cdot {\mathrm{dir}}_{2}\right)a_{2}={\mathrm{dir}}_{1}\cdot \left(p_{2}-p_{1}\right).$$

Similarly, take the partial derivative with respect to $a_{2}$, and after simplifying, we get:

(24) $$\left({\mathrm{dir}}_{2}\cdot {\mathrm{dir}}_{1}\right)a_{1}-\left({\mathrm{dir}}_{2}\cdot {\mathrm{dir}}_{2}\right)a_{2}={\mathrm{dir}}_{2}\cdot \left(p_{2}-p_{1}\right).$$

Combine the Equations (23) and (24) and write them as vector form:

(25) C·a=B,

where

(26) $$C=\left[\begin{array}{cc} {\mathrm{dir}}_{1}\cdot {\mathrm{dir}}_{1} & -{\mathrm{dir}}_{1}\cdot {\mathrm{dir}}_{2}\\ {\mathrm{dir}}_{1}\cdot {\mathrm{dir}}_{2} & -{\mathrm{dir}}_{2}\cdot {\mathrm{dir}}_{2} \end{array}\right],$$

(27) $$a=\left[\begin{array}{c} a_{1}\\ a_{2} \end{array}\right],$$

(28) $$B=\left[\begin{array}{c} \left(p_{2}-p_{1}\right)\cdot {\mathrm{dir}}_{1}\\ \left(p_{2}-p_{1}\right)\cdot {\mathrm{dir}}_{2} \end{array}\right].$$

The solution to the matrix Equation (25) is:

(29) $$\hat{a}=C^{-1}\cdot B.$$

The AOA positioning result is:

(30) $${\hat{p}}_{0,aoa}=\frac{p_{2}+p_{1}+{\hat{a}}_{1}\cdot {\mathrm{dir}}_{1}+{\hat{a}}_{2}\cdot {\mathrm{dir}}_{2}}{2}.$$

Based on the principles of the IWOA described in [41], the following introduction presents the proposed IWOA. The IWOA includes two main strategies: an adaptive update mechanism and a neighborhood search strategy.

The adaptive update mechanism is the core improvement of IWOA. The update of the WOA’s position only considers the current global optimal solution, whereas IWOA introduces the historical best position of the individual $X^{pb}\left(t\right)$ into the update formula. It also uses the linearly decreasing parameter *a* to dynamically adjust the weight between the global best position and the individual best position.

In the early stages of iteration (|A|≥1), the algorithm is in the global search phase, and the update formula is:

(31) $$\begin{array}{cc} X(t+1) & =\frac{1}{2}aX^{pb}\left(t\right)+\left(1-\frac{1}{2}a\right)X^{gb}\left(t\right)-AD_{1} \end{array}$$

(32) $$\begin{array}{cc} D_{1} & =C_{1}X^{pb}\left(t\right)-X\left(t\right) \end{array}$$

(33) $$\begin{array}{cc} C_{1} & =(2r-1)\times a \end{array}$$

(34) $$\begin{array}{cc} a & =2-2\times t/T \end{array}$$

where $X^{pb}\left(t\right)$ is the historical best position of the individual; $X^{gb}\left(t\right)$ represents the current best position; *r* is a random number in [0,1].

In the mid-iteration phase (q<|A|<1, where *q* is a constant threshold), the algorithm enters the local optimization stage, and the update formula is:

(35) $$\begin{array}{cc} X(t+1) & =\frac{1}{2}aX^{pb}\left(t\right)+\left(1-\frac{1}{2}a\right)X^{gb}\left(t\right)-AD_{2} \end{array}$$

(36) $$\begin{array}{cc} D_{2} & =C_{2}X^{\mathrm{gb}}\left(t\right)-X\left(t\right) \end{array}$$

(37) $$\begin{array}{cc} C_{2} & =(2r-1)\times (2-a) \end{array}$$

As *a* decreases, the coefficient (1−a/2) in (35) increases, thereby guiding the population toward a better region.

The neighborhood search strategy aims to enhance the algorithm’s local fine-tuning capability in the later stages. When the algorithm enters the late stage of iteration (|A|≤q), the population conducts small-range perturbation searches around the current global best position:

(38) $$\begin{array}{cc} X(t+1) & =X^{gb}\left(t\right)+n \end{array}$$

(39) $$\begin{array}{cc} n & =(2r-1)\times a\times n \end{array}$$

where *n* represents the step size, and the initial value is set to the upper bound of the population position range.

IWOA relies on an adaptive mechanism to enhance exploration in the early stage to prevent premature convergence, and on neighborhood search to strengthen exploitation and improve accuracy in the later stage, systematically improving the performance of WOA. For the population matrix X with a population size of $N_{p}$, the initialization parameters of IWOA are as follows: the lower bounds of individual coordinates lb, the upper bound of individual coordinates ub, and the initial individual positions $X_{i}$.

(40) $$\begin{array}{cc} M & =k\cdot {∥{\hat{p}}_{0,aoa}∥}_{2}^{2} \end{array}$$

(41) $$\begin{array}{cc} u & =[M,M] \end{array}$$

(42) $$\begin{array}{cc} \mathrm{lb} & ={\hat{p}}_{0,aoa}-u \end{array}$$

(43) $$\begin{array}{cc} \mathrm{ub} & ={\hat{p}}_{0,aoa}+u \end{array}$$

(44) $$\begin{array}{cc} X_{i,j} & ={\mathrm{lb}}_{j}+l\cdot \left[{\mathrm{ub}}_{j}-{\mathrm{lb}}_{j}\right] \end{array}$$

where *M* is the half-length of the searching interval; *k* is the scaling coefficient, typically set to 0.5; lb,ub are lower and upper bound of IWOA initialization; $X\in R^{N_{p}\times 2}$ is an IWOA individual matrix; $X_{i,j},\left(i=1,2,\ldots ,N_{p};j=1,2\right)$ is the coordinate value of the *i*-th whale individual in the *j*-th dimension for each element in X; *l* is a random number following a uniform distribution in the range [0,1].

According to the distance formula, the distance difference of population $X_{i}\left(i=2,\ldots ,N_{p}\right)$ relative to anchor j(j=1,2,…,m) is:

(45) $$\begin{array}{c} r_{i,j}=d_{i,j}-d_{i,1}=\sqrt{{\left(x_{j}-X_{i,1}\right)}^{2}+{\left(y_{j}-X_{i,2}\right)}^{2}}\\ -\sqrt{{\left(x_{1}-X_{i,1}\right)}^{2}+{\left(y_{1}-X_{i,2}\right)}^{2}} \end{array}$$

The residual TDOA for each individual can be expressed as

(46) $$f\left(X_{i}\right)={∥r-d∥}_{2}^{2}.$$

According to (46), the IWOA updates the current best position $X^{gb}\left(t\right)$ and the historical best position $X^{pb}\left(t\right)$. Subsequent iterations are performed multiple times to obtain the optimal positioning solution.

## 5. Performance Evaluation

### 5.1. Simulation Setup

The experimental section of this study consists of two parts. First, to validate the effectiveness of the asynchronous compensation of the algorithm, different values of the pulse interval time *T* at the anchor node—including 1.6 s, 1.0 s, 0.6 s, and 0.3 s—were selected in the simulation. To eliminate random errors in the algorithm, the simulation results were averaged over 1000 independent noise measurements. Second, to verify the superiority of the proposed algorithm in localization, this study employs a hexagonal cellular layout comprising 9 anchor nodes, with the number of anchor nodes selectively varied between 4 and 9 for analysis, where the cellular radius *R* is the value of inter-anchor distance, and the anchor coordinates are configured based on the hexagonal pattern, exemplified by $p_{1}=R\cdot \left[1,0\right],p_{2}=R\cdot \left[-1,0\right],p_{3}=R\cdot \left[1/2,\sqrt{3}/2\right],p_{4}=R\cdot \left[-1/2,\sqrt{3}/2\right],$$p_{5}=R\cdot \left[-1/2,-\sqrt{3}/2\right],p_{6}=R\cdot \left[1/2,-\sqrt{3}/2\right],p_{7}=R\cdot \left[0,\sqrt{3}\right],p_{8}=R\cdot \left[0,-\sqrt{3}\right]$ and $p_{9}=\left[0,0\right]$. A total of 500 targets were randomly placed within a circular area centered at the origin with a radius of *R*, and the positioning results were statistically analyzed. The positioning RMSE is used to characterize the algorithm’s performance.

In addition, Anchors 1 and 2 are specifically designated for AOA measurements. The clock offsets and clock skews between Anchor i(i=2,…,9) and Anchor 1 are modeled as a zero-mean Gaussian distribution with variances of $\sigma_{clock}$ and $\sigma_{f}$, respectively. The TOA measurement error and the AOA measurement error for the angle-measuring anchor nodes follow a zero-mean Gaussian distribution with variances of $\sigma_{toa}$ and $\sigma_{aoa}$, respectively. The channel model assumes line-of-sight propagation conditions. The IWOA is configured with a maximum iteration count of 20 and adaptive weights set to $w_{\max}=0.9$ and $w_{\min}=0.2$. To eliminate random errors inherent in the algorithm, the simulation results were averaged over 500 independent noisy measurements. The experimental setup is implemented on a MATLAB 2024b platform running on a Windows 10 64-bit system.

To demonstrate the superior performance of the proposed optimization algorithm, a systematic comparison is conducted between the localization algorithm based on the IWOA proposed in this study and established methods including the Chan algorithm, Improved Dandelion Algorithm (IDOA) [34], Improved Particle Swarm Optimization (IPSO) [42], Sparrow Search Algorithm (SSA) [43], and Constrained Weighted Least Squares (CWLS) [44]. This comparative analysis aims to validate the effectiveness and advancement of the IWOA-based approach under consistent experimental conditions. Key parameters of the wireless positioning environment are summarized in Table 1 [34,38,41,42,43].

### 5.2. Experimental Comparison

**Figure 5:**
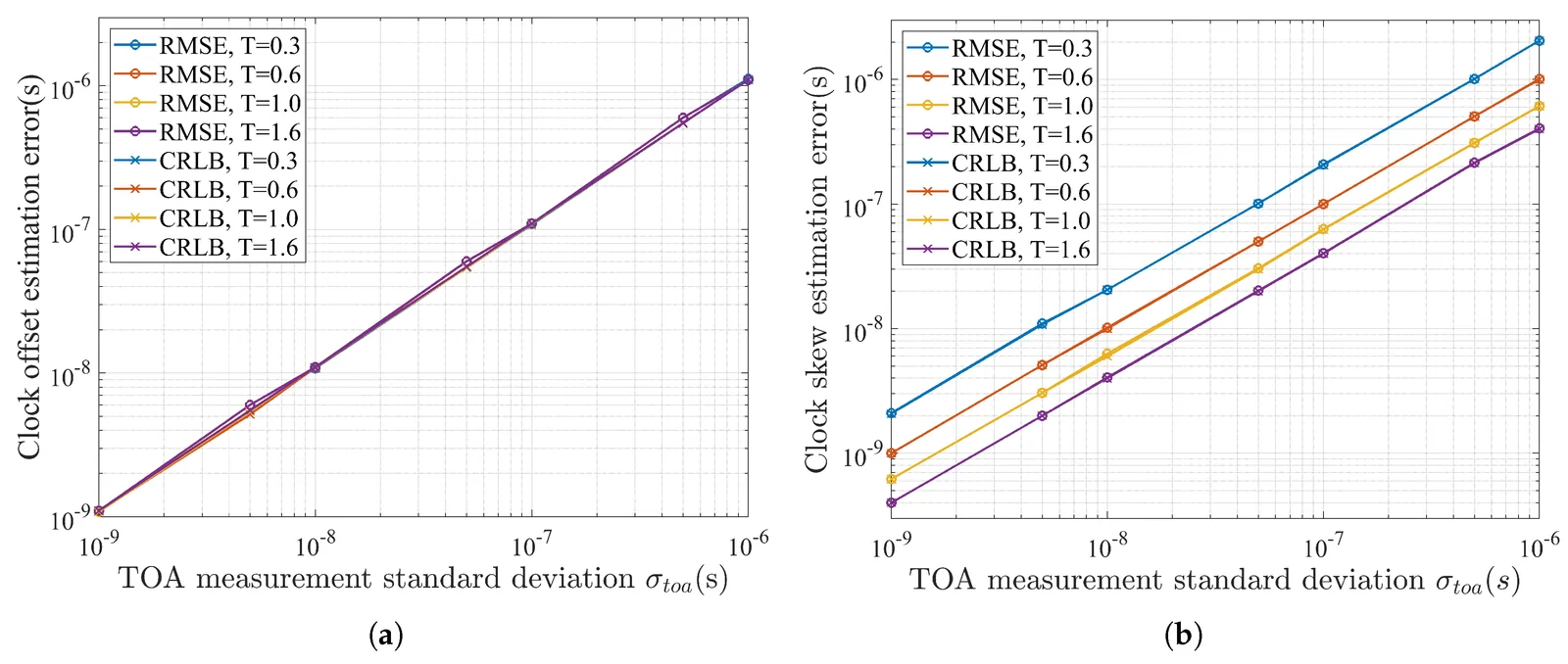
RMSE of clock parameter estimation for different broadcast intervals *T*: (**a**) clock offset estimation; (**b**) clock skew estimation.

As shown in Figure 5a, irrespective of the pulse interval *T*, the root mean square error (RMSE) of the estimated relative clock offset remains unchanged, indicating that *T* does not affect the estimation of the relative clock offset.

From Figure 5b, it can be observed that the RMSE of the estimated relative clock skew decreases as the pulse interval *T* decreases. This behavior arises because the estimated relative clock skew is a weighted average of the TOA measurements over the time span; consequently, shorter pulse intervals result in smaller estimation errors for the relative clock skew.

This study systematically evaluates the performance of various optimization methods within the proposed localization framework from four critical aspects: number of anchor nodes, inter-anchor distances, TOA measurement errors, and angle measurement errors.

**Figure 6:**
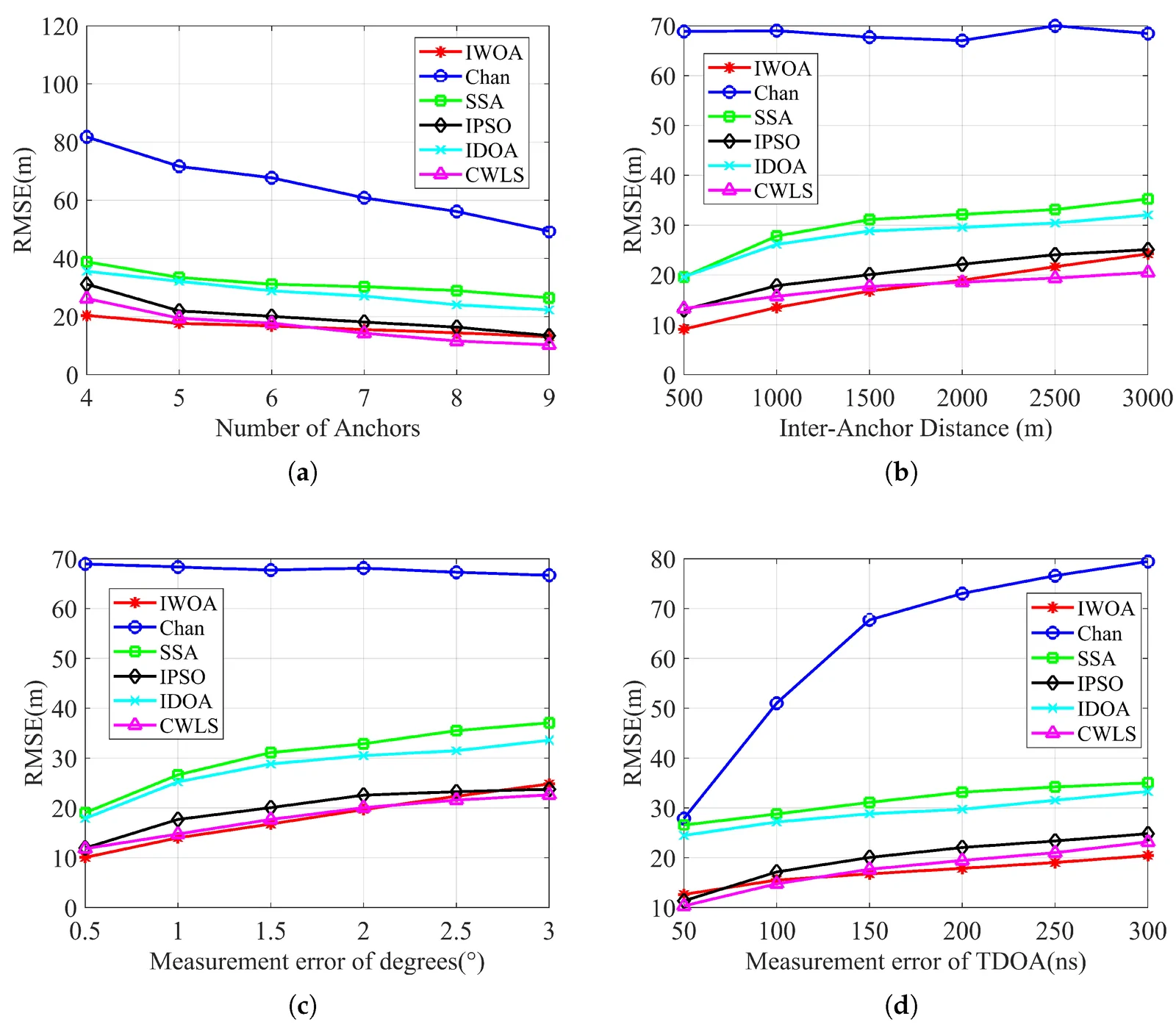
The relationship between localization errors and different parameters: (**a**) number of anchors; (**b**) inter-anchor distances; (**c**) angle measurement errors; (**d**) TOA measurement errors.

With *R* = 1500 m, angular error of 1.5°, and TDOA measurement error of 150 ns, Figure 6a depicts the relationship between positioning accuracy and the number of anchor nodes, comparing the performance of different algorithms. As the number of anchor nodes increased from 4 to 9, the RMSE of all algorithms decreased to varying extents, indicating that additional anchor nodes provide supplementary time difference measurements, thereby enhancing the geometric diversity of the positioning system and improving accuracy. Among them, the reduction in RMSE for the Chan algorithm was the most pronounced, decreasing by approximately 34.3%. IWOA showed its best performance with 4∼6 anchors, but was surpassed by CWLS when the number increased to 7∼9. This is because IWOA initializes its population within a bounded search region determined by the AOA coarse estimate (44). As more anchors are added, the TDOA residual surface becomes increasingly complex, and the fixed search radius derived from the initial AOA estimate may fail to cover the true global optimum, especially when the initial AOA estimate is slightly biased. In contrast, CWLS does not rely on such a bounded initialization; its algebraic solution scales gracefully with additional measurements, effectively exploiting the redundant geometric information to improve accuracy.

Under the configuration of 6 anchor nodes, angular error of 1.5°, and TDOA measurement error of 150 ns, six algorithms were evaluated. Figure 6b illustrates the relationship between the RMSE and the inter-anchor distance. Because the target’s distribution range expands as the radius increases, the geometric accuracy of the target in the chan algorithm hasn’t improved, and the positioning accuracy stays around 79 m. For IWOA, the AOA-based initial positioning error grows with the inter-anchor distance because the same angular error translates into a larger lateral position deviation in a wider geometry. This degraded initial estimate enlarges the search interval and may push the population away from the true minimum of the TDOA residual function. CWLS, by contrast, decouples the TDOA linearization from AOA measurements, thereby preventing the AOA error from propagating into the pseudo-linear equations; hence it maintains more stable performance at larger radius.

In the configuration with 6 anchor nodes, *R* = 1500 m and TDOA measurement error of 150 ns, Figure 6c illustrates the relationship between RMSE and AOA measurement error. As the angle measurement error increases from 0.5° to 3°, the RMSE of all algorithms generally increases, indicating that greater measurement noise degrades positioning accuracy. The Chan algorithm does not utilize AOA measurements; hence its RMSE remains approximately 67 m. At small AOA errors (0.5°–2°), the coarse AOA estimate is sufficiently close to the true position. IWOA capitalizes on this by concentrating its initial population within a tight region around this estimate and then performing a fine-grained neighborhood search, allowing it to effectively locate the global minimum of the nonlinear TDOA residual. CWLS, despite its noise decoupling, remains a linearized approximation; its closed-form solution is subject to the mismatch between the assumed and actual noise covariance matrices, leading to a slight but systematic bias that limits its performance in this low-angle-error regime.

Under the configuration of 6 anchor nodes, *R* = 1500 m and angular error of 1.5°, six algorithms were evaluated. As shown in Figure 6d, when the TDOA measurement error increases from 50 ns to 250 ns, the RMSE of all algorithms exhibits an upward trend. The Chan algorithm yields the highest RMSE and the largest increase (approximately 186%). When the TDOA measurement error is between 50 and 100 ns, the CWLS weight matrix can automatically adjust the weighting between the TDOA residuals and the position error residuals, making the positioning results optimal. However, as the TDOA error increases, the positioning error inevitably grows, and the TDOA residual surface will produce many false local optimal traps. Based on compensating the original TDOA bias through clock parameter estimation, IWOA uses a neighborhood sampling search mechanism to effectively skip these suboptimal solutions caused by noise and converge to the global optimum as much as possible.

## 6. Conclusions

This work tackles the high-precision localization challenge in asynchronous Wireless Sensor Networks (WSNs) by proposing a novel AOA-assisted TDOA localization framework. Its core contributions are threefold. First, we propose an efficient clock parameter estimation method that compensates for asynchronous TDOA errors, establishing a foundation for localization. Second, we develop a fusion algorithm requiring only two angle-measuring anchor nodes alongside TDOA anchor nodes, reducing hardware dependency and cost. Finally, we adopt an Improved Whale Optimization Algorithm (IWOA), which initializes its population using coarse AOA results and iteratively refines the solution by minimizing TDOA residuals, ensuring high accuracy and convergence. Comprehensive simulations validate the algorithm’s effectiveness, showing that the IWOA-based method outperforms benchmarks in most noisy scenarios with smoother convergence. In summary, this research improves the performance of asynchronous WSN localization through integrated clock compensation, data fusion, and optimization, demonstrating significant application potential. Comprehensive simulations also reveal that the proposed IWOA and the CWLS algorithm exhibit complementary strengths. IWOA excels in scenarios with severe TDOA noise, sparse anchor nodes, and moderate geometries, where the non-convex residual surface is riddled with local optima and the asynchronous clock compensation plays a critical role. In contrast, CWLS achieves superior accuracy under well-calibrated conditions with dense anchor nodes, large geometric layouts, and low TDOA measurement errors, benefiting from its algebraically efficient noise-decoupled formulation. This complementary observation suggests that in practical deployments, a strategy could be adopted: applying CWLS for rapid coarse estimation under favourable conditions, and switching to IWOA for robust refinement when noise levels or geometric constraints become severe. Future work will explore 3D localization and non-line-of-sight (NLOS) robustness.

## Figures and Tables

**Table 1:** Simulation Parameters.

Parameter Name	Values
Number of anchor nodes	4∼9
Inter-anchor distance *R*	500∼3000 m
Pulse repetition period *T*	0.3/0.6/1/1.6 s
Variances of clock offset and clock skew: $\sigma_{clock}$ and $\sigma_{f}$	$10^{-6}$ s, $10^{-10}$ $s^{-1}$
Variances of TOA measurement error	50∼250 ns
Variances of AOA measurement error	0.5°∼3°
Number of initial particles	60
Number of maximum iteration	20
IWOA: $w_{\max},w_{\min}$	0.9, 0.2
IDOA: upper limit *U* and lower limit *L*	10,000, 0
IDOA: seed count bounds $m_{max}$,$m_{min}$	120, 18
IPSO: $c_{1},c_{2}$	2.4, 2.4
IPSO: $w_{\max},w_{\min}$	0.9, 0.2
SSA: $ST,R_{2}$	0.6, 0.6

## Data Availability

Data are contained within the article.
